# Effect of Metformin on Antipsychotic-Induced Metabolic Dysfunction: The Potential Role of Gut-Brain Axis

**DOI:** 10.3389/fphar.2019.00371

**Published:** 2019-04-09

**Authors:** Chao Luo, Xu Wang, Hanxue Huang, Xiaoyuan Mao, Honghao Zhou, Zhaoqian Liu

**Affiliations:** ^1^ Department of Clinical Pharmacology, Xiangya Hospital, Central South University, Changsha, China; ^2^ Hunan Key Laboratory of Pharmacogenetics, Institute of Clinical Pharmacology, Central South University, Changsha, China; ^3^ School of Life Sciences, Central South University, Changsha, China; ^4^ National Clinical Research Center for Geriatric Disorders, Xiangya Hospital, Central South University, Changsha, China

**Keywords:** antipsychotic, metabolic dysfunction, metformin, gut-brain axis, gut microbiota, hypothalamus

## Abstract

Antipsychotics are the first-line medications prescribed for patients with schizophrenia or other mental disorders. Cumulative evidence has revealed that metabolic dysfunctions frequently occur in patients receiving antipsychotics, especially second-generation antipsychotics, and these effects may decrease patient compliance and increase health costs. Metformin is an effective pharmaceutical adjuvant for ameliorating antipsychotic-induced metabolic dysfunction (AIMD) in clinical practice. However, the mechanism of the effects of metformin on AIMD remains unclear. The gut-brain axis is a bidirectional communication system between the gastrointestinal tract and the central nervous system and has been associated with many pathological and physiological conditions, such as those related to metabolism. Antipsychotics interact with and have affinity for dopamine receptors and other receptors in the brain, and treatment with these antipsychotics has been shown to influence gut microbiota metabolism and composition, as observed in both animal and human studies. Metformin exerts an antidiabetic effect that is correlated with activation of AMP-kinase in the hypothalamus, and metformin also influences gut flora. Therefore, the gut-brain axis may play a role in the effect of metformin on AIMD. Since no direct evidence is available, this perspective may provide a direction for further research.

## Introduction

Antipsychotics, known for their antagonism of dopamine receptors in the brain, have been widely prescribed for patients with schizophrenia and other mental disorders since the early 1950s (Owen et al., 2016). Second-generation antipsychotics (SGAs) have gradually replaced the first-generation antipsychotics as first-line medications due to their superior therapeutic effects and the lower probability of causing extrapyramidal symptoms. However, patients who receive SGAs are at risk of metabolic dysfunctions, which may induce severe disease [e.g., type 2 diabetes (T2D), cardiovascular disorders, and obesity], decrease patient compliance, and increase health costs (Lochmann van Bennekom et al., 2013; Werner and Covenas, 2014). In clinical practice, metformin has been used as an effective pharmaceutical adjuvant for ameliorating antipsychotic-induced metabolic dysfunction (AIMD) (Wu et al., 2007; Samara et al., 2016; Stroup and Gray, 2018).

To date, the mechanisms of how AIMD can be ameliorated by metformin remain unclear. AIMD is related to many physiological and pathologically altered systems, such as the gut microbiota and many neurotransmitter receptors targeted by antipsychotics (Manu et al., 2015; Reynolds and McGowan, 2017). Metformin, a biguanide, is primarily used for T2D, but is also used in the treatment of obesity and polycystic ovary syndrome (PCOS) (Maruthur et al., 2016). Animal studies have suggested that the effects of both antipsychotics and metformin on appetite are correlated with the effects in the hypothalamus in the brain (Lv et al., 2012; Rojczyk et al., 2015). On the other hand, cumulative evidence has suggested that both antipsychotics and metformin exert their therapeutic effects by influencing gut microbiota metabolism and composition (McCreight et al., 2016; Skonieczna-Zydecka et al., 2018). The neurohumoral communication system between the gastrointestinal tract (GIT) and the central nervous system (CNS) is known as the gut-brain axis, and its dysfunction has been implicated in various endocrine, nutritional, immunological, and psychiatric disorders (Mayer et al., 2015). Therefore, the gut-brain axis may play a role in the effect of metformin on AIMD. Since there is no direct evidence to support this role, in this perspective, we first review the relationships among the gut-brain axis, metformin, and AIMD and then provide a direction for further research.

## The Gut-Brain Axis and Metabolism

There is increasing evidence suggesting that gut microbiota play a vital role in the regulation of the gut-brain axis. In the healthy human gut, *Firmicutes* and *Bacteroidetes* make up the dominant phyla of microbiota, while the proportion of phyla varies in different disease and environmental conditions (Sandhu et al., 2017). Short-chain fatty acids (SCFAs), including butyrate, acetate, and propionate, are predominately generated by *Firmicutes* and *Bacteroidetes* in the colon. SCFAs are known to affect energy homeostasis and regulate glucose, lipid, and cholesterol metabolism in various tissues. In addition, free SCFAs can cross the blood-brain barrier and can influence various regions of the brain. SCFAs exert various physiological effects not only by controlling the release of satiety hormones such as cholecystokinin, glucagon-like peptide 1 and peptide YY in the GIT but also by affecting the expression of anorectic neuropeptides such as the melanocortin precursor proopiomelanocortin (POMC) and neuropeptide Y (NPY) and agouti-related peptide (AgRP) in orexigenic neurons in the hypothalamus (Tolhurst et al., 2012; Frost et al., 2014; Bauer et al., 2016). Hunger and satiety signals are integrated in the brain. Ghrelin, the “hunger hormone,” is secreted in the GIT and regulates appetite by binding to ghrelin receptors in the hypothalamus. Leptin, an adipose-secreted hormone, is opposed by the actions of the hormone ghrelin and regulates energy expenditure by acting on receptors in the hypothalamus. Polymorphisms of the genes coding for these two hormone receptors have been correlated with obesity, T2D, and AIMD (Yang et al., 2012; Puangpetch et al., 2018). Furthermore, activation of the hypothalamic-pituitary-adrenocortical axis may induce changes in the gut flora and intestinal epithelium (Petra et al., 2015). In sum, the bidirectional communication of the gut-brain axis is important for maintaining metabolic homeostasis.

## Antipsychotic-Induced Metabolic Dysfunction and Metformin

### Evidence in Clinical Studies

Metformin treatment has been considered a safe and effective way to ameliorate AIMD. The first report of metformin as an intervention for ameliorating AIMD was conducted in 19 pediatric patients who were receiving antipsychotics, significant weight loss and decreases in body mass index (BMI) were observed after the coadministration (Morrison et al., 2002). Subsequently, two randomized controlled trials (RCTs) confirmed the therapeutic effect of metformin on AIMD in both adolescent and adult patients. One of these studies demonstrated that metformin can prevent olanzapine-induced weight gain and insulin resistance in severe schizophrenia or schizoaffective adults (Baptista et al., 2006). The other RCT was conducted in children and adolescent patients who had more than 10% increases in body weight after at least 1 year of SGA treatment. Metformin treatment was well tolerated and improved weight control, insulin resistance, and abnormal glucose metabolism after 16 weeks of the drug combination (Klein et al., 2006). Similar results were reported in studies of first-episode or drug-naïve schizophrenia patients. Metformin intervention can attenuate antipsychotic-induced weight gain, dyslipidemia, amenorrhea, and insulin resistance (Wu et al., 2008a,b, 2012, 2016).

### Evidence in Preclinical Studies

Similar to humans, rodents receiving antipsychotics presented metabolic dysfunction, while metformin is generally capable of ameliorating AIMD. Coadministration with metformin ameliorated olanzapine- and risperidone-induced weight gain, white fat accumulation, insulin resistance, hyperglycemia, and hyperlipidemia (Adeneye et al., 2011; Boyda et al., 2012; Hu et al., 2014). Interestingly, therapeutic doses of metformin (150 mg/kg) attenuated hepatic insulin resistance in male rats following acute olanzapine treatment, while greater than therapeutic doses of metformin (400 mg/kg) showed an elevation of lactate, a by-product of gut microbiota metabolism (Remington et al., 2015).

## Antipsychotics and the Gut-Brain Axis

### Evidence in Preclinical Studies

The gut microbiota is necessary and sufficient for AIMD. Mice in conventional housing conditions gained more weight after receiving a high-fat diet (HFD) plus olanzapine for 7 weeks than the HFD alone group had gained. In germ-free mice, the HFD plus olanzapine group and HFD alone group showed no significant difference in body weight (Morgan et al., 2014). Likewise, compared to the control group, mice treated with risperidone gained significantly more body weight and reduced energy expenditure. Furthermore, the total resting metabolic rate was decreased in the group that received fecal transplants from the risperidone-treated mice but not in the group that received fecal transplants from vehicle-treated mice (Bahr et al., 2015b). Oral antipsychotic treatment showed an antibiotic-like effect on gut microbiota diversity; for example, olanzapine seemed to decrease alpha diversity, and aripiprazole significantly increased alpha diversity (Morgan et al., 2014; Cussotto et al., 2018). At the phylum level, an increased ratio of *Firmicutes*/*Bacteroidetes* was the most commonly observed effect in rodents receiving SGA treatment (Davey et al., 2012; Morgan et al., 2014; Bahr et al., 2015b; Cussotto et al., 2018). In addition, the abundance of *Actinobacteria* and *Proteobacteria* was decreased in mice after receiving olanzapine in a sex-dependent manner (Davey et al., 2012). The concentration of SCFAs, especially acetate, was increased in the feces of rats with AIMD, which indicated that the structure or function of the microbiota could be partly changed (Cussotto et al., 2018; Kao et al., 2018). Another strong piece of evidence of a relationship between AIMD and the microbiota is that AIMD can be ameliorated or suppressed by other microbiome regulators, such as antibiotics, prebiotics, and probiotics (Davey et al., 2013; Kao et al., 2018).

In addition to dopamine receptors in the brain, antipsychotics also affect a wide range of neurotransmitter receptors, including serotonergic, histaminergic, muscarinic, and adrenergic receptors, which may be correlated with AIMD (Reynolds and McGowan, 2017). The Roman high- and low-avoidance rats showed schizophrenia-like behavioral and physiological characteristics. Compared to vehicle-treated rats, body weight, blood glucose, and dopaminergic receptor expression in the cortico-mesolimbic system were higher in the olanzapine-treated Roman high-avoidance rats; in contrast, the Roman low-avoidance rats showed no differences in these measures. The discrepancy was due to the Roman high-avoidance rats having elevated central dopaminergic sensitivity, which indicated that dopamine receptor expression is related to olanzapine-induced weight gain (Evers et al., 2017). The serotonin 2C receptor has been implicated in many neurological and biological processes, including the regulation of food intake, body weight, and glucose metabolism. Unlike wild-type mice with olanzapine treatment, *HTR2C*-knockout mice showed no significant differences in body weight, food intake, blood glucose or insulin levels compared with these measures in the vehicle-treated group. In addition, mice that received lorcaserin, a specific serotonin 2C receptor agonist, also showed a suppression of olanzapine-induced hyperphagia and weight gain (Lord et al., 2017). Histamine receptors were the most studied neurotransmitter receptor in association with AIMD. Olanzapine-treated female rats showed a significant reduction in H1 receptor binding density in the ventromedial nucleus and a reduction in histamine H1 mRNA expression in the arcuate nucleus and ventromedial nucleus, which correlated with olanzapine-induced increases in body weight and food intake (Han et al., 2008). Similar results were observed in risperidone-treated rats, but not with aripiprazole or haloperidol treatment (Han et al., 2008; Lian et al., 2015). Furthermore, compared with controls, olanzapine increased AMPKα activation in the hypothalamus. This increase was accompanied by significantly upregulated NPY and H1 receptor mRNA expression and downregulated POMC mRNA expression, which correlated with appetite, glucose and fatty acid uptake activation and oxidation (Lian et al., 2014). Coadministration of betahistine, an H1 receptor agonist and an H3 receptor antagonist, was capable of significantly ameliorating olanzapine-induced weight gain and reducing feeding efficiency, which confirmed the role of the histamine receptor in AIMD (Deng et al., 2012).

### Evidence in Clinical Studies

The gut-brain axis seems to play an important role in neuropsychiatric disorders, especially in neurodegenerative diseases (Westfall et al., 2017; Nguyen et al., 2018). Bahr et al. (2015a) conducted a cross-sectional study and a prospective study in male psychiatric children and adolescents with risperidone treatment. Compared to the control group, patients who received chronic risperidone administration exhibited significant weight gain accompanied by an increased diversity of gut microbiota and ratio of *Firmicutes*/*Bacteroidetes*, while the phyla ratio gradually increased over the course of treatment (Bahr et al., 2015a,b). In another cross-sectional study, BMI was higher and microbiome diversity was significantly lower in SGA-treated bipolar disorder patients than in non-SGA-treated individuals. The abundance of *Lachnospiraceae* was preferentially increased, while the abundance of *Akkermansia* was decreased, in the SGA-treated group compared with the non-SGA-treated group (Flowers et al., 2017). The latest study was conducted in first-episode and drug-naïve SCZ patients. Compared to healthy controls, RIS-treated patients had significant metabolic dysfunction, increased abundance of *Bifidobacterium* spp. and *Escherichia coli* and decreased abundance of the *Clostridium coccoides* group and *Lactobacillus* spp. (Yuan et al., 2018). These results provided direct evidence linking the gut microbiome to AIMD in humans; however, replicated studies are needed in the future.

Satiety-related hormones, such as ghrelin and leptin, as well as some metabolic parameters, were altered in AIMD patients. A recent meta-analysis demonstrated that blood leptin levels were increased, while blood ghrelin levels were decreased, in patients with AIMD (Potvin et al., 2015; Goetz and Miller, 2018). Furthermore, polymorphisms of the satiety-related hormone receptor genes (*GHRL*, *LEP*, and *LEPR*) showed significant associations with AIMD in clinical studies (Gregoor et al., 2009; Wu et al., 2011; Yang et al., 2012). In addition to these genes, polymorphisms of genes encoding for receptors that antipsychotics have affinity for, neurodevelopmental modulators, and neuroendocrine pathway-related proteins have been investigated for association with AIMD *via* pharmacogenetic studies. The *HTR2C* rs3813929 polymorphism was the first found functional single-nucleotide polymorphism associated with antipsychotic-induced weight gain in Chinese schizophrenia patients (Reynolds et al., 2002). However, due to the diverse allele frequencies in different ethnic groups, discrepant results have been observed in studies with different populations. Recently, Brandl et al. (2016) conducted the first genome-wide association study (GWAS) in patients with AIMD, and rs9346455 of the *OGFRL1* gene was significantly associated with weight gain in the Clinical Antipsychotic Trials of Intervention Effectiveness (CATIE) samples after various antipsychotic treatments. The other GWAS of AIMD was performed in the Chinese population, and the *PTPRD* rs10977144 polymorphism showed the strongest association with weight gain (Yu et al., 2016). A systematic review and meta-analysis of pharmacogenetic studies from around the world revealed that polymorphisms of *HTR2C*, *MC4R*, and *LEP* genes had consistent associations with AIMD, while another study showed that only polymorphisms of *HTR2C* consistently showed associations with AIMD in the Chinese population (Zhang et al., 2016; Luo et al., 2019).

## Metformin and the Gut-Brain Axis

### Evidence in Preclinical Studies

Cabreiro et al. (2013) first demonstrated that metformin increased the lifespan of *Caenorhabditis elegans* co-treated with *Escherichia coli* by altering microbial metabolism, suggesting that metformin has the potential to affect the intestinal microbiome in mammals. Indeed, cumulative evidence has revealed that metformin exerts a therapeutic effect by inducing significant changes in gut microbial metabolism and microbiota composition (Lee and Ko, 2014; Zhang et al., 2015; Lee et al., 2018). In healthy mice, metformin increased the abundance of *Verrucomicrobia* and *Bacteroidetes* and decreased the abundance of *Firmicutes* and *Proteobacteria* (Ma et al., 2018). The genera *Akkermansia* and *Lactobacillus* were significantly enriched in HFD-fed mice coadministered metformin compared to their levels in the control group (Shin et al., 2014; Lee et al., 2018). Both genera resulted in a significant improvement in the body weight, insulin, glucose and lipid profiles, which was subsequently confirmed in a model of gut microbiota transfer from HFD-fed rodents with metformin treatment into recipient peers (Shin et al., 2014; Zhou et al., 2016; Bauer et al., 2018; Lee et al., 2018).

AMPK (AMP-activated protein kinase), mainly expressed in liver and brain, plays a role in cellular energy homeostasis, affects glucose and fatty acid uptake and oxidation. Metformin not only mediates duodenal AMPK-dependent neuronal signaling to influence the gut-brain axis but also penetrates the blood-brain barrier to act on the CNS directly (Lv et al., 2012; Duca et al., 2015). In diabetic rats, oral metformin treatment reduced food intake by influencing orexigenic peptides, for example, through decreased mRNA expression of NPY and AgRP but not POMC and activation of signal transducer and activator of transcription 3 (STAT3) but not AMPK (Lv et al., 2012). Although the food intake was reduced in rats following intracerebroventricular injection of metformin, hypothalamic POMC but not NPY mRNA levels were elevated, and both phosphorylation of STAT3 and AMPK were increased (Lee et al., 2012). In addition to neuropeptides, metformin also influences other neurotransmitter receptors in the hypothalamus. The 5-HT_3_ receptor is expressed throughout the central and peripheral nervous systems and mediates a variety of physiological functions, such as GIT motility (Michel et al., 2005). Due to the structural similarity of metformin with selective agonists of the 5-HT_3_ receptor, metformin-induced 5-HT_3_ receptor-independent release of serotonin was observed in murine neuroblastoma N1E-115 cells (Cubeddu et al., 2000). Metformin improved leptin and insulin sensitivity *via* elevated receptor expression in the hypothalamus, which contributed to the anorectic effects (Aubert et al., 2011; Malin and Kashyap, 2014; Tang et al., 2016). In addition, a recent study in baboons and macaques demonstrated that metformin regulated metabolic parameters by acting on the pituitary gland through multiple molecular pathways (Vazquez-Borrego et al., 2018).

### Evidence in Clinical Studies

Treatment with metformin has long been known to have side effects, including diarrhea, nausea, and abdominal pain, which indicates that metformin may exert a therapeutic effect not only in the liver but also in the GIT. The side effects were associated with an increase in relative abundance of *Escherichia-Shigella* spp. in healthy volunteers treated with metformin (Elbere et al., 2018). Compared to healthy controls, patients treated with metformin partially reversed T2D-induced *Subdoligranulum* and *Akkermansia* dysbiosis in samples from multiple countries. Moreover, the comparison of gut microbiota composition between metformin-treated and untreated T2D patients has revealed that metformin significantly increased the relative abundance of *Escherichia* spp. and reduced the relative abundance of the *Intestinibacter* genus (Forslund et al., 2015). Likewise, metformin enriched mucin-degrading *Akkermansia muciniphila* and several SCFA-producing bacteria while decreasing the abundance of the *Intestinibacter* genus in drug-naïve patients with T2D and a large cohort of Colombian T2D patients (de la Cuesta-Zuluaga et al., 2017; Wu et al., 2017). In addition to its effect on gut microbiota, metformin was capable of regulating bile acid turnover, enhancing gut-related peptide secretion, and regulating intestinal glucose uptake and glucose homeostasis, which indicated an underlying mechanism of metformin’s ability to ameliorate metabolic dysfunctions (Brønden et al., 2017; Borg et al., 2018; Sun et al., 2018).

Metformin affects the hypothalamus-pituitary-thyroid axis to influence hormone secretion and metabolic parameters. In women with PCOS, metformin improved glucose homeostasis accompanied by decreased serum luteinizing hormone and thyrotropin levels, which indicated that metformin treatment may have an impact on pituitary activity (Billa et al., 2009; Krysiak and Okopien, 2015). Another clinical study demonstrated that T2D- or antipsychotic-induced hyperprolactinemia was significantly suppressed in women receiving metformin (Krysiak et al., 2016, 2018). Furthermore, a pharmacogenetic study revealed that the rs2815752 polymorphism of *NEGR1* (gene encoding the neuronal growth regulator 1 protein) was correlated with long-term weight loss in diabetic patients treated with metformin (Delahanty et al., 2012).

## Conclusion

**Figure 1:**
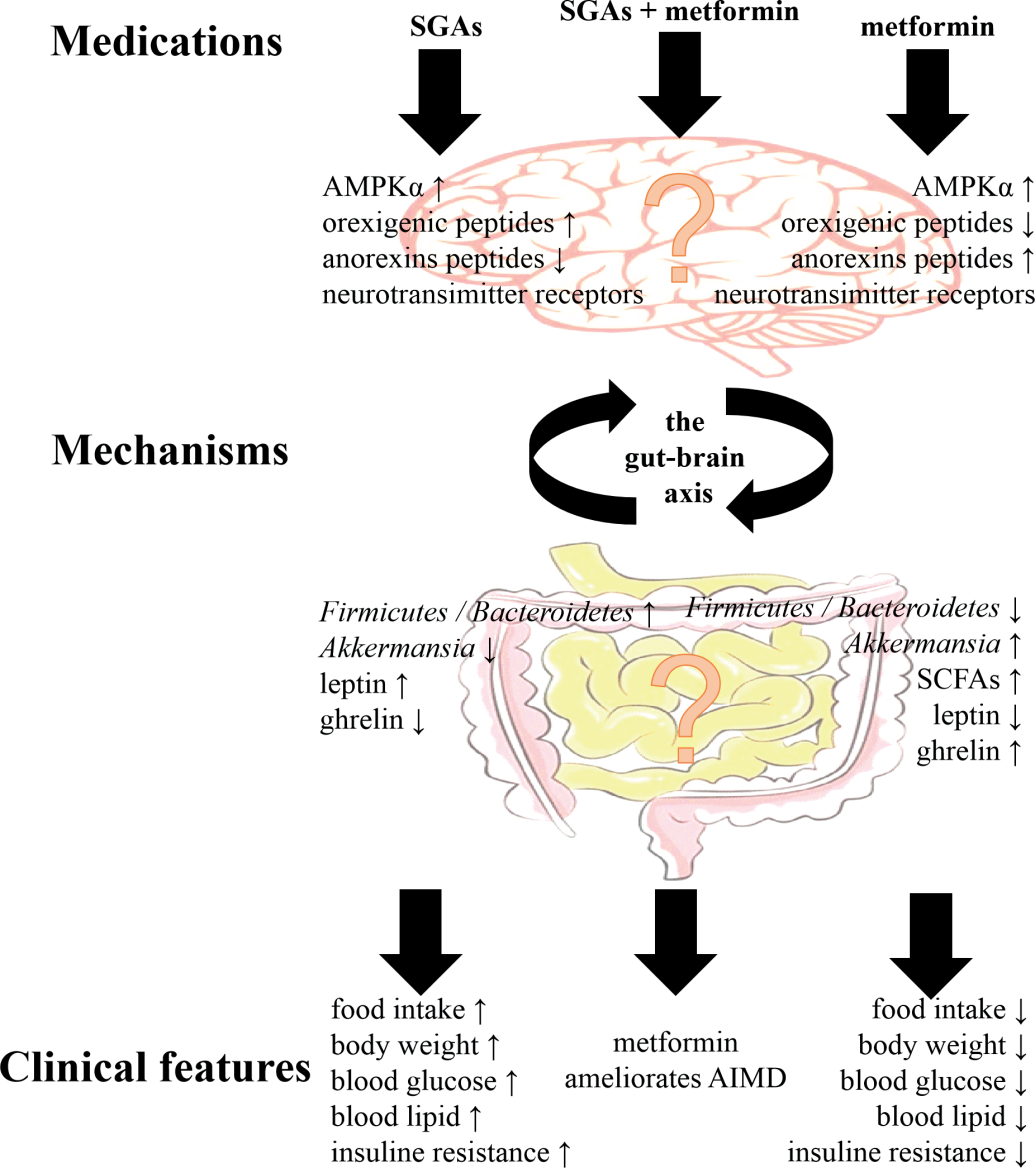
The main clinical features of second-generation antipsychotics (SGAs) and metformin, and their main mechanisms *via* the gut-brain axis. Although metformin ameliorates antipsychotic-induced metabolic dysfunction (AIMD), the mechanisms *via* the gut-brain axis still remained to be investigated. AMPKα, adenosine monophosphate-activated protein kinase α; SCFAs, short-chain fatty acids.

Preclinical and clinical studies have demonstrated that treatment with antipsychotics and metformin had effects on the gut microbiota and the brain. Although the mechanism of the effects of metformin on AIMD remains unclear, there are some clues in the regulation of the gut-brain axis (Figure 1). The most important connections between metformin and AIMD in the gut microbiota were the mucin-degrading bacteria *A. muciniphila* and several SCFA-producing bacteria, such as the phyla *Firmicutes* and *Bacteroidetes*. The gut microbiota were was influenced during AIMD, and these changes included a decrease in *A. muciniphila* and an increase in the ratio of *Firmicutes*/*Bacteroidetes* (Skonieczna-Zydecka et al., 2018). Metformin enriched the abundance of *A. muciniphila* and lowered the ratio of *Firmicutes*/*Bacteroidetes* when exerting its therapeutic effects (McCreight et al., 2016). In contrast, antipsychotics are known for their affinity for many neurotransmitter receptors that are capable of activating hypothalamic AMPK signaling, increasing levels of orexigenic peptides such as NPY and AgRP and decreasing levels of anorexins (Lian et al., 2016). With metformin treatment, reduced NPY and AgRP expression and enhanced POMC expression in the hypothalamus have been observed (Duca et al., 2015). In addition, metformin ameliorated blood leptin elevations and ghrelin decreases in AIMD, which are two major hormones that affect the hypothalamus to regulate appetite (Malin and Kashyap, 2014). Although the gut-brain axis may play a theoretical role in the effect of metformin on AIMD, the relationship still remains to be investigated.

## Author Contributions

CL, XW, and HH did literature research. XM, HZ, and ZL gave conceptual advice and critically revised the manuscript. CL designed and wrote the manuscript. All authors contributed to the conceptualization and approved the final version of this manuscript.

### Conflict of Interest Statement

The authors declare that the research was conducted in the absence of any commercial or financial relationships that could be construed as a potential conflict of interest.

## Abbreviations

AgRP: Agouti-related peptide
AIMD: Antipsychotic-induced metabolic dysfunction
BMI: Body mass index
CNS: Central nervous system
GIT: Gastrointestinal tract
GWAS: Genome-wide association study
HFD: High-fat diet
NPY: Neuropeptide Y
PCOS: Polycystic ovary syndrome
POMC: Proopiomelanocortin
RCT: Randomized controlled trial
SCFA: Short-chain fatty acid
SGA: Second-generation antipsychotic
STAT3: Signal transducer and activator of transcription 3
T2D: Type 2 diabetes.
